# Molecular characterization of *Helicobacter pylori* isolated from Nile Tilapia (*Oreochromis niloticus*) and fish handlers

**DOI:** 10.1186/s12917-023-03819-6

**Published:** 2023-11-29

**Authors:** Asmaa Gaber Mubarak, Hanan H. Abd-Elhafeez, Hams M. A. Mohamed

**Affiliations:** 1 Department of Zoonoses, Faculty of Veterinary Medicine, South Valley University, Qena, 83523 Egypt; 2 Department of Cell and Tissues, Faculty of Vet. Medicine, Assiut University, Assiut, 71526 Egypt; 3 Department of Microbiology, Faculty of Veterinary Medicine, South Valley University, Qena, 83523 Egypt

**Keywords:** Antibiotic resistance, Fish, *H. Pylori*, Human, Phylogeny

## Abstract

**Background:**

*Helicobacter pylori* is a worldwide pathogen that affects both animals and humans with a wide environmental distribution, causing serious health problems in humans. This research has timely addressed the topic of new sources of *H. pylori* infection, which is currently a global issue, especially in developing countries. For this purpose, 115 Tilapia fish, 50 freshwater samples, and 88 fish-handlers’ stool samples were investigated for the presence of *H. pylori* in Qena Governorate, Egypt. The applied techniques were antigen screening tests, culturing, and molecular methods through *ure*C gene amplification, and *16 S rRNA* characterization.

**Results:**

*Helicobacter pylori* was detected in 7.83%, 14%, 4.35%, and 12% of the investigated fish and water samples by culture and PCR methods, respectively. Out of the total studied participants, 40 tested positive for *H. pylori* when screened by stool antigen test, of which 35 (39.77%), and 31 (35.23%) were confirmed by conventional and molecular techniques, respectively. The Fisher’s exact test has shown a statistically significant correlation between *H. pylori* infection, sex, and age as risk factors, while the association was insignificant concerning the residence. Males contracted the infection at a higher rate than females (48.08% and 16.67%, respectively). Also, *H. pylori* infection rate was the highest among fish-handlers aged 36–45 years old (46.67%), followed by the 26–35 years old age group (39.53%). With regard to the residence, a higher occurrence rate was recorded in the rural (36.07%) than the urban population (33.33%). *Helicobacter pylori* isolates harbored the highest antimicrobial resistance against ampicillin (100%), metronidazole (95.24%), while the least antimicrobial resistance was recorded against levofloxacin (21.43%), and clarithromycin (26.20%). The phylogenetic analysis revealed a high degree of homology between the isolates selected from Tilapia fish, freshwater, and fish-handlers.

**Conclusions:**

Our data emphasized the role that fish and freshwater play in disseminating *H. pylori* infection as one of the diseases that has a significant public health issue.

## Background

*Helicobacter pylori* infection has a significant public health impact on human health in both developed and developing countries, as it is classified by the WHO as a bacterial carcinogen. Additionally, it is commonly associated with chronic infections, and peptic ulcers [1]. *Helicobacter pylori* is a microaerophilic Gram negative spiral shaped bacteria belonging to the family *Helicobacteraceae* that particularly colonize the human gastric epithelium and have been isolated from the gastrointestinal tract of many animals, causing one of the most common infections worldwide [2, 3].

The genus *Helicobacter* comprises over 35 species, of which *H. pylori* is the most important in terms of human health [4]. The global prevalence of *H. pylori* is around 50%, and the infection with this microbe may be of greater importance in developing countries where 70–90% of the population is infected with *H. pylori*, compared to 25–50% in industrialized countries [5]. In Egypt, *H. pylori* infection is estimated to be 60–80% by several studies [6, 7]. *Helicobacter pylori* infection has been linked to the emergence of different gastro-intestinal diseases, such as gastric ulcers and gastric cancer [8].

As the bacterium can survive in animals’ stomachs, food products such as milk, meat, and vegetables are crucial in the spread of *H. pylori* infection [9]. On the other hand, water plays a critical role in the transmission of the infection, either as an environmental reservoir or through fecal-oral transmission, especially in unhygienic conditions. The association between *H. pylori* antibodies and those produced during the infection with two known waterborne pathogens; hepatitis A virus, and giardia; has been documented, so, the possibility of *H. pylori* to be waterborne has been raised [10]. This bacterium can naturally live in aquatic habitats such as lakes, rivers, drinking water supplies, and coastal marine ecosystems, from which sea food may become contaminated [11].

Fish is a highly nutritious food containing proteins, unsaturated fatty acids, minerals, and vitamins, so it’s widely marketed and consumed [12]. Nevertheless, the slightly acidic pH of fish meat, besides its chemical composition, makes it highly susceptible to deterioration and contamination by different biological agents [13]. Interestingly, *H. pylori* was detected in Tilapia in numerous aquatic environments at various localities, unlike other fish species, even those taken from the same water source [14]. Nile Tilapia (*Oreochromis niloticus*) is among the most popular and cheap fish for Egyptians, and has the ability to withstand in low oxygen, and high ammonia levels, so it can survive in poor environmental conditions [15], making it more susceptible to contamination by several biological contaminants; in particular bacteria; as *H. pylori* [14, 16].

The fecal-oral route of transmission of *H. pylori* has more important implications than the oral-oral route, as it occurs in food and water supplies after fecal contamination. Several studies have proven *H. pylori* to be a food-borne pathogen because of its microbiological and epidemiological characteristics. Many individuals harbor this organism and never develop any clinical symptoms; however, others may develop signs of nausea, vomiting, diarrhea, abdominal pain, and fever. In some cases, some complications may occur, such as meningitis, abortion, Guillan-Barré syndrome, Reiter’s syndrome, cancer, and even death [17].

As *H. pylori* is a fastidious, slow-growing bacterium, and as the exposure to different environmental conditions changes its characteristic from rod shape to coccoid form, many authors indicated that the culture method is very difficult. While successful isolation and identification of *H. pylori* can be affected by a number of factors; including the clinical specimen quality, the time between sampling and culture, and improper transport conditions; nevertheless, the culture method is still considered the gold standard. In addition, *H. pylori* stool antigen test is rapid, accurate, and has a high sensitivity and specificity for detection of active and recent *H. pylori* infection [18]. Moreover, the specificity and sensitivity of anti-*H. pylori* antibody test are 79–90%, and 76–84%, respectively. Molecular methods are commonly used to detect or confirm *H. pylori* to get more accurate diagnostic performance [19], but they have not been accepted for routine testing.

The antibiotic resistance of *H. pylori* represents a great problem because it affects the success of eradication therapy, and therefore, its treatment has proven to be incredibly challenging. Over the past two decades, there has been an increased antibiotic resistance rate of *H. pylori* all over the world [20, 21]. Therefore, *H. pylori* has been identified by the WHO as one of the 20 pathogens that represent the greatest harm to human health [17]. The resistance of *H. pylori* to metronidazole, clarithromycin, levofloxacin, amoxicillin, and tetracycline has been reported. While *H. pylori* antibiotic resistance varies regionally, there is no consistent report on whether it is related to age and gender [22]. Due to *H. pylori* being a significant human pathogen, there is an ongoing need to identify additional environmental sources of this bacterium as potential routes for its dissemination and transmission to humans. Therefore, this study was designed to ascertain the presence of *H. pylori* in fish, natural environmental water, and humans. As well as, to describe some risk factors may related to human infection as sex, age and residence. Furthermore the relationship between the isolates and their antibiotic resistance.

## Materials and methods

### Ethical declaration

The Research Ethics Committee Regulations of South Valley University, Qena, Egypt (VM/SVU/23(2)-28) were adhered in conducting this protocol. This study participants provided their informed consent. All procedures were carried out in compliance with the applicable rules and regulations. The study was conducted in accordance with the ARRIVE (Animals in Research: Reporting In Vivo Experiments) criteria [23].

### Study design and sampling

Between February 2021 and November 2022, this study was conducted in Qena Governorate, Egypt, located approximately 576 km from Cairo (26°10′12′′N, 32°43′38′′E). A total of 115 Tilapia fish were purchased from various markets and immediately transported to the laboratory for investigation in sterile ice boxes. For each fish, the intestinal content was promptly obtained using a sterile scalpel blade and mixed with buffered peptone water (BPW) in sterile glass. Additionally, 50 water samples were collected from different freshwater streams in the governorate (River Nile branches). In a sterile glass (polypropylene), 250–500 ml of each sample was collected, transported to the laboratory, and stored at 4 °C for analysis.

In the same context, 88 fresh stool samples were collected from fish handlers who exhibited symptoms of gastritis such as stomach pain, frequent burping, bloating, and unexplained weight loss. The samples were collected in sterile, sealed cups, labeled, and promptly transported to the laboratory in ice boxes for immediate investigation. Sociodemographic characteristics of the participants, including sex, age, and residence, were documented.

### Antigen screening test

For the direct detection of *H. pylori* antigen in stool samples from fish-handlers, the stool antigen test (SAT) is utilized which is a qualitative immunochromatographic method. Using an on-site *H. pylori* antigen quick test cassette (CTK Biotech, USA), 50 mg of each sample was examined for the presence of *H. pylori* antigen following the manufacturer’s instructions.

### Isolation and identification of ***H. pylori***

The seropositive stool samples, as well as fish intestinal content, and water samples were subjected to isolation and identification of *H. pylori* according to Sainsus et al. [24], with slight modifications as follows: Aseptically, 10 mL of Brain Heart Infusion broth (BHIB) with a 5% *Helicobacter* selective supplement was added to a sterile test tube with 1 mL of each homogenized sample of human feces and fish intestinal content. A 0.22 μm Millipore filter membrane was used to filter water samples. After that, each membrane was submerged for 1 h in 2 mL of tryptic soy broth [25]. Then, 1 mL of TSB was diluted with a 5% *Helicobacter* selective supplement, and added to the sterile test tube containing BHIB. With the aid of an active gas producing kit (5% H_2_, 5% CO_2_, 5% O_2_, and 85% N_2_), inoculated test tubes were incubated at 37 °C in a microaerophilic environment in an anaerobic jar and monitored daily.

For 96 h, the turbidity of the broth was tracked, and then it was streaked using the plating out method on Columbia agar with 5% defibrinated sheep blood supplemented with *Helicobacter* selective supplement. The streaked plates were incubated at 37 °C for 3–5 days under microaerophilic conditions in an anaerobic jar as previously mentioned. The morphology of the suspected colonies, microscopic examination, and biochemical tests (oxidase, catalase, urease, nitrate reduction, and hippurate hydrolysis tests) were used to identify the colonies.

### DNA extraction

Extraction of *H. pylori* DNA from suspected colonies was done using QIAGEN QIAamp DNA mini kit instructions.

### Molecular identification of ***H. pylori*** using the ***ure***C (***glm***M) gene

According to Van Zwet et al. [26] (Table 1), the *ure*C (*glm*M) gene was the target of a PCR that was used to identify *H. pylori*. The PCR mixture contains;12.5 µL the Emerald Amp Max PCR Master Mix (Takara, Noji-higashiKusatsu, Japan), 1 µL of each primer, 4.5 µL of water, and 6 µL of DNA template. The amplification reaction’s thermal profile included an initial denaturation at 95 °C for 5 min, 30 cycles of 94 °C for 3 min, 94 °C for 1 min, 62 °C for 1 min, and 1 min at 72 °C, and a final extension at 72 °C for 5 min. After electrophoresis, distinct bands at 417 bp were visualized.

**Table 1:** Primer sequences of *Helicobacter pylori* used in this study

Gene	Sequence	Amplicon size	Reference
***ure*** **C gene**	EHC-V:(5′-CCCTCACGCCATCAGTCCCAAAAA-3′) EHC-L:(5′-AAGAAGTCAAAAACGCCCCAAAAC-3′)	411 bp	Van Zwet et al. [26]
***16 S rRNA***	F27-AGAGTTT G ATC MTGGCTCAG R1492-TACGGTACC TTGTTACGACTT	1500 bp	Chèneby et al. [27]

### Amplification and sequencing of the ***16 S rRNA*** gene

In order to partially amplify and sequence the *16SrRNA* gene, six isolates were randomly selected from freshwater, Tilapia fish, and fish handlers (two of each) (Table 1). The amplification condition and PCR components were performed according to Chèneby et al. [27]. The PCR products were separated using 5 V/cm gradient electrophoresis on 1.5% agarose gel (Applichem GmbH, Darmstadt, Germany) at room temperature. 20µL of the products were put into each gel slot. A gel documentation system (Alpha Innotech, Biometra, Göttingen, Germany) used to photograph the gel. The QIA quick PCR Product extraction kit (Qiagen, Valencia, CA) was used to purify the PCR products. For the sequence reaction, Bigdye Terminator V3.1 cycle sequencing kit (Perkin-Elmer) was used, and then was purified using Centrisep spin column. DNA sequences were obtained by Applied Biosystems 3130 genetic analyzer (HITACHI, Tokyo, Japan).

### Phylogenetic analysis

Using the MegAlign module from the Lasergene package (version 7), the phylogenetic tree was created. By comparing our *16SrRNA* sequences to those found in the database, MEGA6 software was used to create a neighbor-joining phylogenetic tree [28].

### Phenotypic detection of antibiotic resistance

According to Ranjabar et al. [29], antimicrobial susceptibility testing for seven therapeutically useful antibiotics was completed using the Kirby-Bauer disc diffusion method on Muller-Hinton supplemented with 5% defibrinated sheep blood and incubated at 37 °C for 48 h in a microaerophilic atmosphere (85% N_2_, 10% CO_2_, and 5% O_2_) in accordance with the Clinical Laboratory Standards Institute’s (CLSI, [30]) recommendations. Ampicillin (AM, 10), metronidazole (MET, 5), erythromycin (ER, 5), clarithromycin (CLR, 2), amoxicillin (AMX, 10), levofloxacin (LEV, 5), and rifampicin (R, 30) (Thermo Fisher Scientific, Basingstoke, United Kingdom) were evaluated for their concentrations in micrograms per disk (mcg/disk). The Clinical Laboratory Standards Institute (CLSI, [30]) recommendations were followed while measuring zone diameters.

### Statistical analysis

SPSS version. 28 was used for the statistical analysis of the study’s data. Through Geisser-Greenhouse’s epsilon calculation, the connection between the positive *H. pylori* infection and the sources of the investigated samples was determined. Fisher’s exact test was computed to establish the risk factors, and a 95% confidence interval (CI) was calculated. For the variations in prevalence rates, p-values under 0.05 were regarded as significant.

## Results

**Fig. 1:**
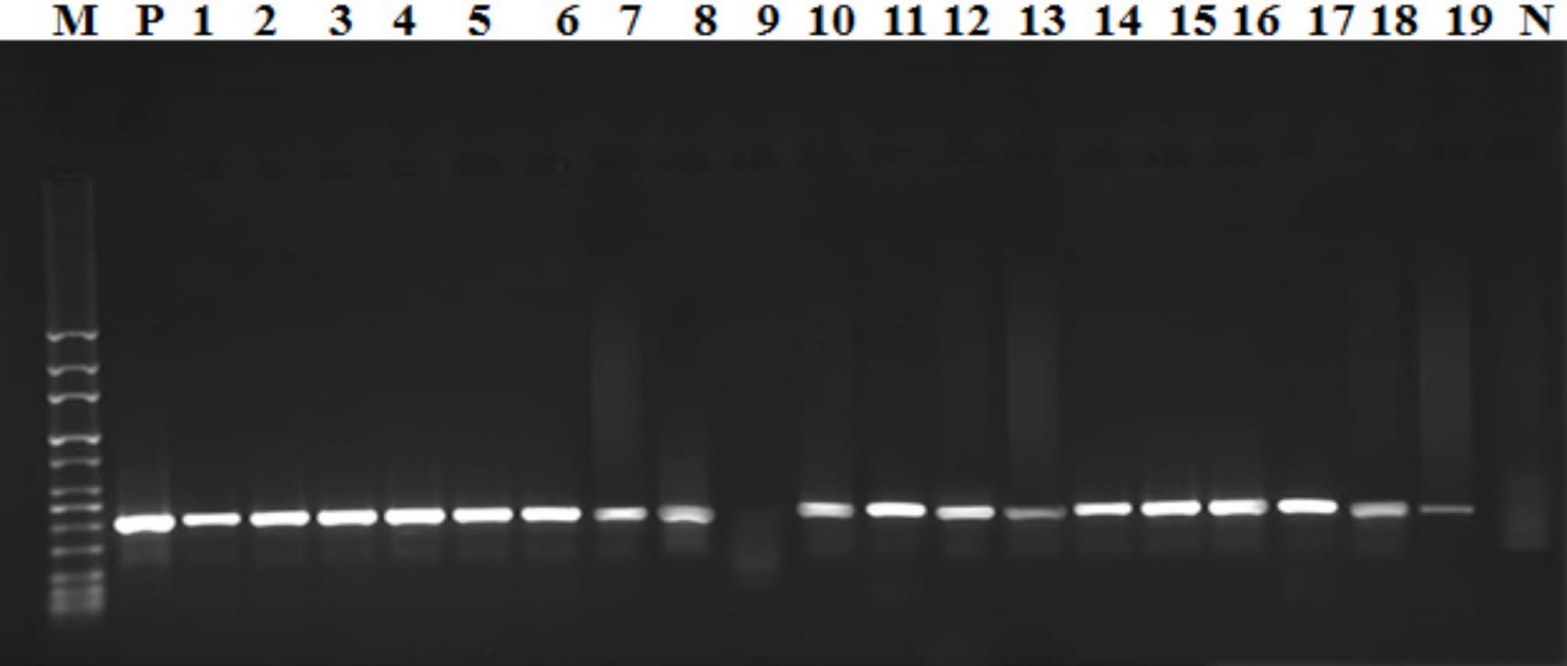
Agarose gel electrophoresis of *ure*C (*glm*M) gene showing bands at 411 bp. Lane M: Marker (DNA ladder 100 bp), Lane P: positive control, Lane (1, 2, 3, 4, 5, 6, 7, 8, 10, 11, 12, 13, 14, 15, 16, 17, 18, 19): positive *ure*C (*glm*M) gene, Lane 9: negative isolate, and Lane N: negative control

In this study, *H. pylori* was found to have a total incidence of 20.16% using conventional methods, and 16.60% using PCR, as presented in Table 2; Fig. 1. As an unusual source of infection, the current study revealed a culture prevalence of *H. pylori* in Tilapia fish of 7.83% (9 out of 115) collected from various freshwater streams. These isolates displayed a negative nitrate reduction biochemical profile and positive results for catalase, oxidase, and urea hydrolysis tests. When confirmed using the *ure*C gene, only five samples (4.35%) tested positive. Out of the 50 freshwater samples, seven tested positive for *H. pylori* using conventional method, while six were positive using PCR. Regarding the human stool samples, 40 of the 88 fish-handlers initially tested positive for *H. pylori* through an antigenic test, and their samples were subsequently cultured, resulting in 35 positive samples (39.77%). However, when these isolates were further confirmed using the *ure*C gene, only 31 of them (35.23%) were confirmed as *H. pylori*. No significant difference was found between human infection with *H. pylori* and the contamination levels of Tilapia fish and freshwater streams as potential sources of infection (*P* = 0.2679).

**Table 2:** *Helicobacter pylori* in the examined samples

The examined samples	No. of examined samples	Positive *H. pylori*		Geisser-Greenhouse’s epsilon
		Conventional methods	PCR	
		No. (%)	No. (%)	R^2^ (*P*)
**Tilapia fish**	115	9 (7.83)	5 (4.35)	0.1175 (0.2679)
**Freshwater streams**	50	7 (14)	6 (12)	
**Fish-handlers**	88	35 (39.77)	31 (35.23)	
**Total**	253	51 (20.16)	42 (16.60)	

A total of 88 fish handlers agreed to participate as volunteers in this study, with 52 being male and 36 being female, as shown in Table 3. The incidence of *H. pylori* in human stool samples differed significantly (*P* = 0.0479) with 48.08% among males compared to 16.67% among females. Participants’ ages ranged from 16 to over 45 years and were classified into four groups. Among these age groups, participants aged between 36 and 45 years had the highest *H. pylori* infection rate (46.67%), followed by the 26–35 age group (39.53%). These two age groups were significantly associated with the rate of *H. pylori* infection (95% CI, *P = 0.0498).* The age group of ˃ 45 years exhibited an incidence of 30.77%, while, those aged 16–25 had the lowest infection rate (17.65%). The residences of the participants were distributed as follows: 27 urban and 61 rural. Rural residents had the highest prevalence of *H. pylori* (36.07%, 22/61) compared to urban residents (33.33%, 9/27) with no significant difference (*P* ˃ 0.9999).

**Table 3:** * H. pylori* isolated from humans in association with risk factors

Variable		No. of examined cases	Positive *H. pylori* No. (%)	95% CI *P*
**Sex**	Male	52	25 (48.08)	0.0479^*^^
	Female	36	6 (16.67)	
**Age**	16–25 y	17	3 (17.65)	0.0498^*^
	26–35 y	43	17 (39.53)	
	36–45 y	15	7 (46.67)	
	˃ 45 y	13	4 (30.77)	
**Residence**	Urban	27	9 (33.33)	> 0.9999^
	Rural	61	22 (36.07)	

^ = Fisher’s Exact Test

* Significant value

**Table 4:** Antibiotic resistance profile of *H. pylori* isolates

Sources of isolates	No	Antimicrobial resistance profile	MAR index
**Tilapia fish**	1 2 3 4 5	AM, MET, RIF, ER AM, RIF, AMX, CLR AM, RIF, AMX, ER, CLR AM, MET, RIF, ER AM, MET, AMX	0.571 0.571 0.714 0.571 0.429
**Fresh water streams**	1 2 3 4 5 6	AM, MET, AMX, ER AM, MET, RIF, ER AM, MET, RIF, ER, CLR AM, MET, AMX, ER AM, MET, RIF, AMX, ER, CLR AM, MET, RIF, AMX, ER	0.571 0.571 0.714 0.571 0.857 0.714
**Human diarrhea**	1 2 3 4 5 6 7 8 9 10 11 12 13 14 15 16 17 18 19 20 21 22 23 24 25 26 27 28 29 30 31	AM, MET, RIF, AMX, ER, LEV AM, MET, AMX, CLR AM, MET, AMX, ER AM, MET, RIF, ER, LEV AM, MET, RIF, AMX, ER AM, MET, RIF, ER AM, MET, RIF, AMX, ER, CLR, LEV AM, MET, RIF, AMX AM, MET, RIF, ER AM, MET, RIF, AMX, ER AM, MET, RIF, AMX,CLR, LEV AM, MET, RIF, AMX, ER AM, MET, RIF, AMX, LEV AM, MET, RIF, AMX, ER, CLR AM, MET, RIF, AMX, ER AM, MET, RIF, ER, LEV AM, MET, RIF, AMX AM, MET, RIF, AMX, ER, CLR, LEV AM, MET, RIF, AMX AM, MET, RIF, AMX, ER AM, MET, RIF, AMX AM, MET, RIF, ER, LEV AM, MET, RIF, AMX, ER AM, MET, RIF, ER AM, MET, RIF, AMX, ER, CLR AM, MET, RIF, AMX,ER AM, MET, RIF, AMX, ER AM, MET, RIF, AMX, ER AM, MET, RIF, AMX, ER, LEV AM, MET, RIF, AMX, ER AM, MET, RIF, AMX, CLR	0.857 0.571 0.571 0.714 0.714 0.571 1 0.571 0.571 0.571 0.857 0.714 0.714 0.857 0.714 0.714 0.571 1 0.571 0.714 0.571 0.714 0.714 0.571 0.857 0.714 0.714 0.714 0.857 0.714 0.714
		Average 0.687	

MAR index = No. of resistance / Total No. of tested antibiotics

**Fig. 2:**
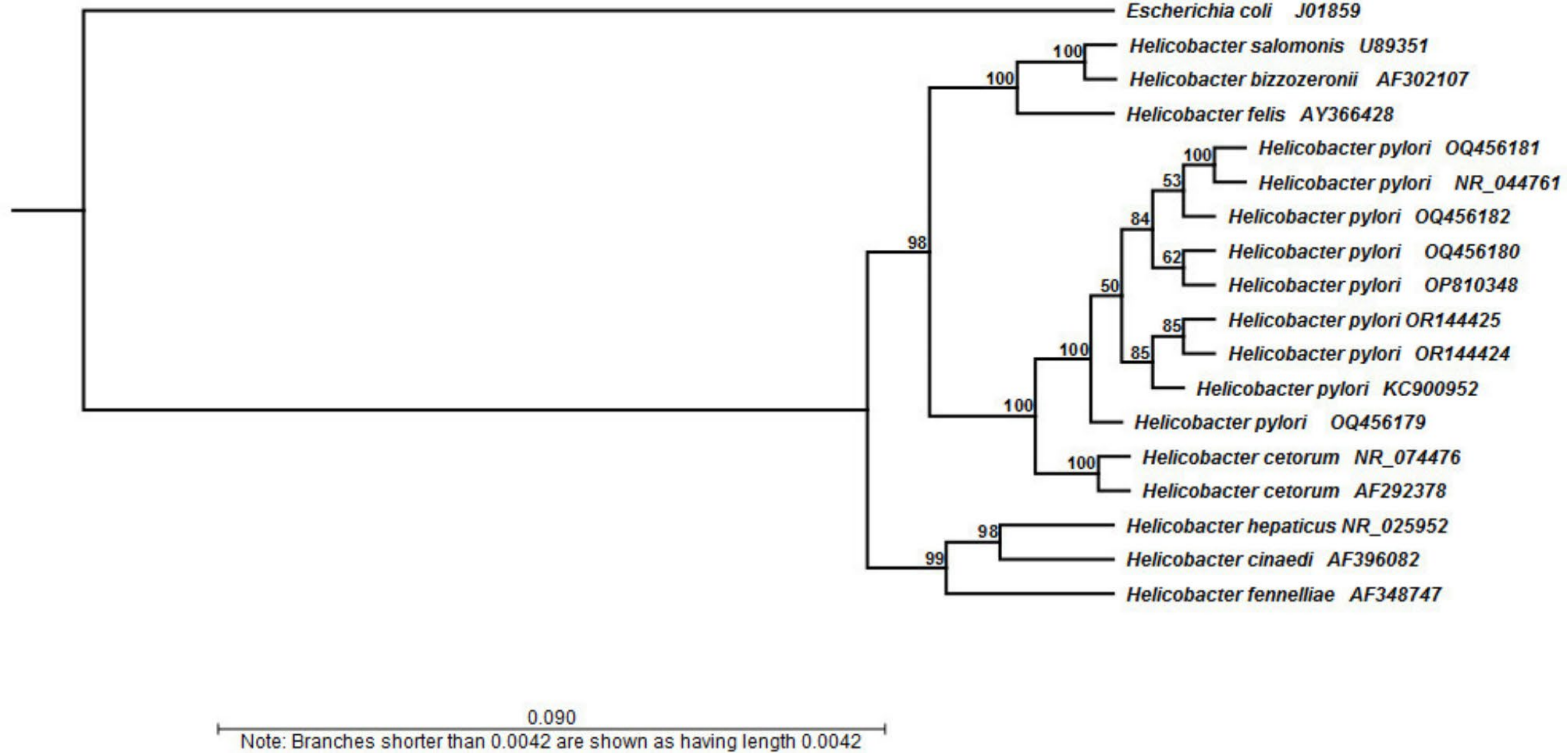
Neighbor-joining phylogenetic tree of the obtained *H. pylori* isolates based on *16 S rRNA* gene sequence

The sequencing results of partially amplified *16SrRNA* for six isolates, selected randomly from fish intestinal contents (OQ456179, OQ456180), freshwater streams (OR144424, OR144425), and fish-handlers’ stool (OQ456181, OQ456182), confirmed that our isolates were indeed *H. pylori*. The phylogenetic analysis revealed a high level of identity (99–100%) among our isolates, despite their diverse sources. Additionally, our isolates were grouped with reference strains from Genbank such as *H. pylori* NR044761, *H. pylori* OP810348, and *H. pylori* KC9009952 with identities of 97.3–97.4%, 100%, and 99.5–100%, respectively (Fig. 2).

**Fig. 3:**
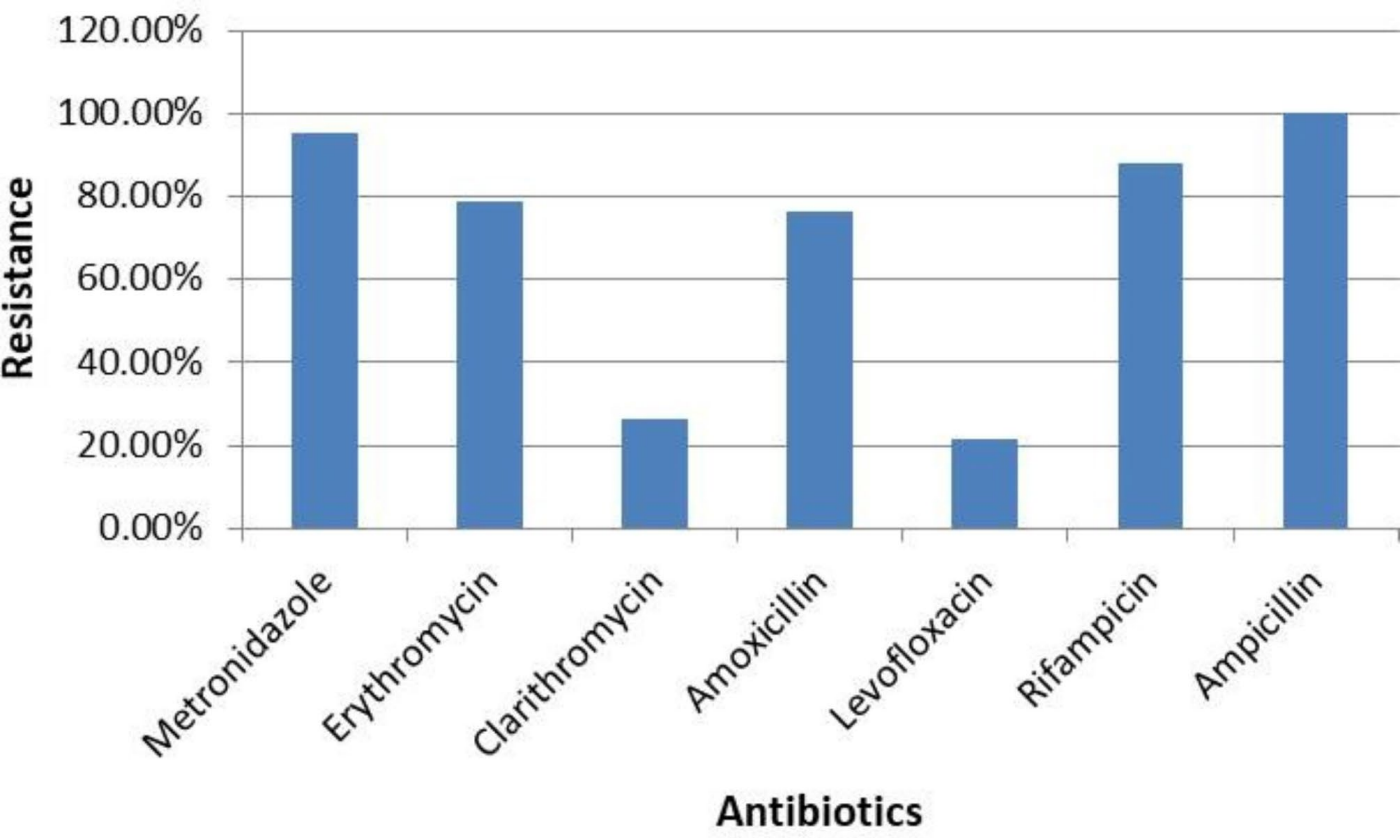
Frequency of antimicrobial resistance of *H. pylori* isolates

Of the 42 *H. pylori* isolates tested for antibiotic resistance, the results are provided in Fig. (3). These results revealed complete resistance to ampicillin (100%), followed by metronidazole (95.24%, 40/42), and rifampicin (88.10%, 37/42). Closely related resistance rates were observed for erythromycin (78.57%, 33/42), and amoxicillin (76.19%, 32/42), while a low frequency of resistance to levofloxacin (21.43%, 9/42), and clarithromycin (26.20%, 11/42) was reported. As shown in Table (4), the MAR index ranged from 0.571 to 1 in the obtained*H. pylori* isolates, with the highest index value of 1 found in one isolate of fish-handlers’ stool. It’s worth noting that most of *H. pylori* isolates were multi-drug resistant (MDR) to at least three classes of antibiotics as observed in Tilapia fish and human isolates.

## Discussion

*Helicobacter pylori* has emerged as one of the most concerning human pathogens associated with water, consequently, it has the potential to contaminate marine food products. While few studies have addressed this issue, the current study tried to evaluate the prevalence of *H. pylori* in fish, freshwater streams, and fish handlers, along with an evaluation of some the risk factors associated with the infection. Culturing of Tilapia fish samples revealed an incidence of *H. pylori* at 7.83%, and after molecular confirmation through the use of the *ure*C gene, only five isolates (4.35%) were identified as *H. pylori*. Wang et al. [31] found that although bacteriological culture is considered as the gold standard for infection diagnosis, *H. pylori* poses particular challenges. It requires additional resources for growth, a microaerophilic environment, and extended incubation, making it difficult to conduct timely sensitivity studies. The technical difficulties associated with *H. pylori* culture are evident, highlighting the need for simpler and quicker methods to identify *H. pylori* and assess its resistance. Molecular techniques have yielded promising results in this regard [32].

Many researchers have endorsed the use of the *ure*C (*glm*M) gene as a genetic marker for *H. pylori* identification, particularly in clinical samples [33]. Additionally, Wongphutorn et al. [34] have recommended the combination of several target genes for *H. pylori* detection, such as *ure*C and *16 S Rrna* sequencing, to enhance diagnostic performance.

Few authors have emphasized fish as a source of *H. pylori*; Abdel-Moein et al. [14] could detect the organism in 1.9% of Tilapia fish. Pina-Pérez et al. [35] identified *H. pylori* in various marine food products, including shellfish. They verified the potential connection between the presence of *H. pylori* in seawater and the role of contaminated seafood as means for *H. pylori* to enter the food chain. Moreover, Cortés-Sánchez [12] has proved that Tilapia fish are a vehicle for the transmission of *H. pylori*.

*Helicobacter pylori* is a significant emerging pathogen associated with water, raising significant concerns for the scientific community [36]. Al-Sulami et al. [25] were the first to identify 10 isolates of *H. pylori* in water through biochemical tests. Our study has confirmed the presence of 7 isolates from 50 freshwater samples (14%) using cultural and biochemical methods. When confirmed by PCR, six isolates were obtained, reducing the rate to 12%. Twing et al. [37] were also able to detect *H. pylori* in freshwater samples. In Basrah Governorate, Iraq, water samples have been contaminated by *H. pylori* at an incidence of 2.76% [25]. Meanwhile, in Egypt, two isolates from tap water and ground water (one from each) were identified as *H. pylori* through direct detection of the *ure*C gene [38]. Other authors all over the world succeeded in detecting the organism in various types of water using different techniques. Ranjbar et al. [29] revealed that 3% of drinking water samples had *H. pylori* through a combination of the culture method and the PCR technique based on *16 S rRNA*. Also, *H. pylori* could be detected in influent water samples in Colombia at a percentage of 22.6% [39]. Besides that, Farhadkhani et al. [11] detected *H. pylori* with a high frequency (36%) in wastewater samples, stressing that fecal pollution of water and other environmental samples plays an important role in *H. pylori* infection transmission. On the other hand, Gholami-Borujeni et al. [1] reported that 24% of water samples collected from urban areas, and 18% from home water treatment devices in Iran tested positive for *H. pylori*.

Differences in *H. pylori* prevalence in water could be attributed mainly to water characteristics including hardness, alkalinity, temperature, and pH, in addition to the use of various methods for detection. Two hypotheses are supported by the presence of *H. pylori* DNA in river water, well water, wastewater, and surface and shallow groundwater: either *H. pylori* is a waterborne organism or water may become contaminated by the fecal-oral route [25].

*Helicobacter* infection is a global zoonotic infection with a high incidence, especially in Egypt [40]. In some cases, *H. pylori* persists in the human host without causing illness. However, it has traditionally been linked to various gastrointestinal illnesses due to induction of local and systemic inflammation [41]. Although human infection with *H. pylori* has declined in many countries as a result of advances in treatment and improved living standards, it still poses a significant risk in developing nations with overcrowded living conditions, inadequate sanitation, and unsafe drinking water. Therefore, investigating the prevalence rate in a large-scale population is crucial, and various diagnostic methods for *H. pylori* are available. In our study, we examined 88 fish-handlers for the presence of *H. pylori* using the stool Ag test, which yielded 40 positive samples (45.45%). This method has been endorsed by many authors as rapid, reliable, and non-random [42]. A closely related result was obtained by Khoder et al. [43] who recorded 41% of healthy asymptomatic residents in the United Arab Emirates as *H. pylori* positive. While higher detection rates were observed in the Egyptian patients stools by Salem et al. [7] and Shaaban et al. [44] at 73.7% and 74.8%, respectively, using the rapid antigen test, On the other hand, lower results were reported by Hassan et al. [45], Almashhadany and Mayass [46], and Seid et al. [47] as 25, 30.4, and 18.45%, respectively. This disparity in *H. pylori* prevalence could be attributed to the sociodemographic status of the investigated population, geographical variations, and hygienic measures. On the other hand, the results of the stool antigen test can be impacted by the heterogeneity of the antigens, the serological procedures used, and the amount of antigen present in the stool [48].

After culturing stool samples, the incidence rate of *H. pylori* has decreased to 39.77% (35/88). A compatible result was obtained by Fabricio-Guaman et al. [49] who obtained a lower incidence rate of *H. pylori* in patients by culture method (36%), than by antigenic screening test (50%). Contrarily, Abu-Gharbia et al. [50] recorded an incidence rate of *H. pylori* in patients attending Assiut University Hospital and some laboratories at incidence rates of 76.9% and 86.1% by stool antigen and culture methods, respectively.

PCR-based techniques are crucial for fast and accurate identification of *H. pylori* infections among patients [51]. So, in the present study, the *ure*C gene was amplified to confirm that the isolates yielded a 35.23% incidence rate. Another point of this investigation includes the occurrence of *H. pylori* in fish-handlers according to some risk factors such as sex, age, and residence. According to the current study results, a statistically significant correlation between sex and the occurrence of *H. pylori* infection has been found. Based on molecular detection, males were more infected (25, 48.08%) compared with females (6, 16.67%). Contrarily, a slightly higher infection rate was detected in females (19.55%) than in males (16.44%) by using an antigen screening test [46]. Through molecular and serological studies, which were done by Castro et al. [52], Mehesin et al. [42], and Shaaban et al. [44], there was no significant higher infection rate in females than males, as 14.6, 11.2 and 55.5, 44.5 and 79.7, 65.5%, respectively. Moreover, Rasheed et al. [53] and Wang et al. [22] were in agreement with our result that males were more prone to *H. pylori* infection than females. This could be explained by the greater exposure of males to environmental factors as a result of working outside homes for longer periods than females. According to the latest studies on the worldwide prevalence of *H. pylori*, there are no variances in *H. pylori* infection based on sex [54].

This study included 88 fish-handlers at ages ranging from 16 to more than 45 years old; the vast majority of the participants were 26–35 years old. The highest level of *H. pylori* infection was reported significantly in the 36–45 year old age group (46.67%), followed by the 26–35 year old age group (39.53%), and then the age group of ˃45 years. This can be explained by the nature of this age; it is the age of working and so more contact with contaminated sources occurs. In our study, the youngest age group (16–25 years) was less susceptible to *H. pylori* infection (17.65%); this result was proportionate with that obtained by Shah et al. [55], who recorded the lowest infection rate in the youngest age group 11–30 years (19.8%). Nevertheless, different results were recorded by previous studies, which were conducted by Khoder et al. [43], who found that *H. pylori* infection increased extremely with age, and Shaaban et al. [44], who recovered the highest infection rate in the over 50 years age group (87.3%), followed by the 15–50 years age group (76.8%). On the other hand, Mehesin et al. [42] demonstrated that there was no correlation between the prevalence of *H. pylori* infection and age.

Along the same lines as other studies in terms of residence, our findings indicated a higher prevalence of *H. pylori* infection in rural residents (36.07%) than in urban residents (33.33%), with no significant difference. Salem et al. [7] stated that farmers are more likely to contract *H. pylori* infection (82.1%) than non-farmers (64.9%). Mehesin et al. [42] recorded an infection rate among rural and urban populations of 60.7 and 39.3%, respectively. Also, Shaaban et al. [44] declared that the infection was more prevalent in those living in rural areas (88.5%) than in those settling in urban areas (51.7%). This may be due to the socio-economic status of the population, low educational level, poor hygienic conditions, and inadequate living resources. These factors facilitate the acquisition of infection, as well as contact with animals, which are considered a significant source of infection, as various studies have shown. This supports the theory that it is a zoonotic disease.

The *16SrRNA* gene displays a high level of functional and evolutionary homology within all bacteria, making it a common choice for *H. pylori* detection [56]. In this study, the sequencing of the *16SrRNA* gene played a major role in confirming our isolates as *H. pylori*, a finding supported by Gong and El-Omar [57]. The phylogenetic analysis revealed that our isolates were closely related to reference strains found in the human gastrointestinal tracts. This raises concerns about the potential role of the fecal-oral route in the transmission of this microorganism.

Increasing rates of *H. Pylori* resistance are associated with a number of clinical issues; the gradual increase in the number of antibiotic-resistant *H. pylori* strains and their diverse resistance profiles makes treatment more challenging and necessitates more pertinent research. Furthermore, chromosome-encoded mutations are most frequently attributed to *H. pylori’s* antibiotic resistance, along with other factors such as membrane permeability alterations, efflux systems, and biofilm formation [58]. Our investigated *H. pylori* isolates were all resistant to at least three families of antibiotics. In addition, the highest resistance was seen against ampicillin, metronidazole, rifampicin, erythromycin, and amoxicillin, which are the best choices for *H. pylori* treatment. This may be related to the indiscriminate use of these antibiotics, which are unfortunately frequently prescribed and in excess of recommended dosages. The antibiotics resistance mechanism can be explained by limiting drug uptake and inactivation, modifying the drug target, or active drug efflux [59]. In addition, there are other mechanisms such as mutational adaptations, acquisition of genetic material or alteration of gene expression leading to resistance to almost all antibiotics [60].

All of the isolated strains were resistant to ampicillin (100%); Ranjbar et al. [29] reported a somewhat lower rate of resistance at 75%. Our *H. pylori* isolates exhibited a high resistance rate to metronidazole (95.24%). Abdoh et al. [61] and Metwally et al. [62] reported a resistance rate of 100%. Various rates of metronidazole resistance were obtained by several authors, including Ranjbar et al. [29] (62.5%), Brigitte et al. [63] (97.9%), Tang et al. [64] (90.6%), Mégraud et al. [65] (58.6%), and Shrestha et al. [66] (69%). The high resistance to this antibiotic may be attributed to its widespread use for many infectious diseases and its cheap price.

Resistance of *H. pylori* isolates to rifampicin, erythromycin, and amoxicillin was reported in various studies. Ranjbar et al. [29] recorded the same resistance rates against erythromycin and amoxicillin (62.5%). In the line with our high resistance level to rifambicin (88.10%), Metwally et al. [62] detected a 90% resistance rate. In contrast, *H. pylori* strains recovered by Mégraud et al. [67] were highly susceptible to amoxicillin, with only a 1.2% resistance rate being detected.

Among our strains, the lowest resistance rate was recorded for levofloxacin (21.43%), which aligns with its recommended use in the treatment of *H. pylori* infection. Furthermore, several studies have reported zero resistance to levofloxacin [61, 63]. In contrast, Tang et al. [64], Mégraud et al. [65], and Metwally et al. [62] reported resistance rates of 28.2%, 17.6%, and 20% to levofloxacin, respectively.

Among our strains, the macrolides, clarithromycin is the most important agent for eradicating *H. pylori* infection. We detected a relatively low level of resistance to clarithromycin in our study (26.20%). Similar results were detected by Shrestha et al. [66] (26%), while Brigitte et al. [63], Hofreuter et al. [68], and Mégraud et al. [67] recorded lower rates of 13.75%, 10.9%, and 22.2%, respectively. In contrast, Abdoh et al. [61] identified high resistance levels at 47% and [64] (44.4%), respectively. The resistance to Clarithromycin in *H. pylori* may be attributed to its misuse in the treatment of various respiratory infections. Furthermore, a high MAR index of up to 1 was observed in our isolates, indicating multiple drug resistance. These findings were corroborated by Rotchell et al. [69], who reported that when the MAR is more than 0.2, it means that the high-risk contamination source is the place where antibiotics are frequently used.

## Conclusions

The presence of *H. pylori* in fish is still unclear, despite its status as a major waterborne disease. To the best of our knowledge, this work is precedent in assessing the prevalence of *H. pylori* in fish, freshwater sources, and fish-handlers. The use of the *ure*C gene as a genetic marker plays a major role in identifying and confirming the presence of *H. pylori* bacteria in our samples. In addition, The creation of a phylogenetic tree revealed genetic relatedness among the isolates. This data supports the hypothesis of a zoonotic route of *H. pylori* transmission. Therefore, there is a need to raise public awareness about stopping the discharge of sewage into waterways to prevent water and fish pollution. Since our study identified a significant level of antibiotic resistance, increased efforts from health officials are necessary to combat it. It is also crucial to implement health education programs and promote strict hygiene practices within households.

## Data Availability

Requests for materials should be addressed to A.G.M and H.M.A.M. Accession numbers and [PERSISTENT WEB LINK]”. OQ456179. https://www.ncbi.nlm.nih.gov/nucleotide/OQ456179.1?report=genbank&log$=nucltop&blast_rank=2&RID=HD49290W013. OQ456180. https://www.ncbi.nlm.nih.gov/nucleotide/OQ456180.1?report=genbank&log$=nucltop&blast_rank=1&RID=HD4G6TMH013. OR144424. https://www.ncbi.nlm.nih.gov/nucleotide/OR144424.1?report=genbank&log$=nucltop&blast_rank=1&RID=HD4X692S01N. OR144425. https://www.ncbi.nlm.nih.gov/nucleotide/OR144425.1?report=genbank&log$=nucltop&blast_rank=1&RID=HD4S86VZ016. OQ456181. https://www.ncbi.nlm.nih.gov/nucleotide/OQ456181.1?report=genbank&log$=nucltop&blast_rank=2&RID=HD4G6TMH013. OQ456182. https://www.ncbi.nlm.nih.gov/nucleotide/OQ456182.1?report=genbank&log$=nucltop&blast_rank=1&RID=HD4MA8RJ013.

## Abbreviations

*H. pylori*: *Helicobacter pylori*
*ure*C: urease C gene
*glm*M: phosphoglucosamine mutase
CLSI: Clinical & Laboratory Standards Institute
MAR: Multiple antibiotic resistance
